# Consumption of Animal-Source Protein is Associated with Improved Height-for-Age *z* Scores in Rural Malawian Children Aged 12–36 Months

**DOI:** 10.3390/nu11020480

**Published:** 2019-02-25

**Authors:** Yankho Kaimila, Oscar Divala, Sophia E. Agapova, Kevin B. Stephenson, Chrissie Thakwalakwa, Indi Trehan, Mark J. Manary, Kenneth M. Maleta

**Affiliations:** 1 School of Public Health and Family Medicine, College of Medicine, University of Malawi, Private Bag 360, Blantyre 3, Malawi; ykaimila1@gmail.com (Y.K.); odivala@yahoo.com (O.D.); cthakwalakwa@yahoo.com (C.T.); manarymj@wustl.edu (M.J.M.); 2 Washington University School of Medicine, Department of Pediatrics, Campus Box 8116, St. Louis, MO 63110, USA; shtepaz@gmail.com (S.E.A.); kbstephe@gmail.com (K.B.S.); itrehan@wustl.edu (I.T.); 3 Lao Friends Hospital for Children, P.O. Box 873, Luang Prabang 06000, Lao PDR

**Keywords:** animal source food, stunting, acute malnutrition, EED, 24-h dietary recall, dietary diversity, PDCAAS, legume complementary food

## Abstract

Linear growth faltering, caused by insufficient diet, recurrent infections and environmental enteric dysfunction (EED), continues to plague young children in low- and middle-income countries (LMICs). Diets in LMICs are primarily plant based, and thus have poor-quality protein and low levels of essential micronutrients. The aim of this study was to assess the association of the type and protein quality of food consumed with stunting, EED and acute malnutrition in children aged 6–36 months in Limera and Masenjere, two rural Southern Malawian communities. This is a secondary analysis of two randomized controlled trials that tested the effects of common bean and cowpea flour on stunting in children aged 6–36 months. We used data from two interactive 24-h dietary recalls conducted 12 weeks after enrolment into each trial. Food intakes were compared between the regions using Chi-square and Student’s *t*-test. There were 355 children that participated in the dietary recalls. The diets of children were of poor quality, but the children from Limera consumed more fish (54% vs. 35%, *p* = 0.009) and more bioavailable protein (26.0 ± 10.3 g/day vs. 23.1 ± 8.1 g/day, *p* = 0.018, respectively) than children in Masenjere. Food type and protein quality were not associated with any of the outcomes except an association between animal protein consumption and improvement in height-for-age *z* scores in children aged 12–36 months (*p* = 0.047). These findings support the notion that animal-source food (ASF) consumption in this vulnerable population promotes linear growth.

## 1. Introduction

Childhood stunting is the most prevalent form of undernutrition in the world, with a 22% (151 million) global prevalence in children under five years old. Presently, 66% of stunted children live in low- and middle-income countries (LMICs) [1]. In Malawi, 37% of children under five years old are stunted [2]. Stunting reduces a child’s physical, immunological and cognitive capacities throughout his/her lifetime and is estimated to account for 21% of all disability-adjusted life years (DALYs) in children [3]. Some factors that are associated with stunting include household and family food insecurity, household food allocation, abbreviated breast feeding, complementary feeding with low energy and nutrient density of traditional foods, reduced dietary diversity and frequent infections such as diarrhea, malaria, respiratory infections and environmental enteric dysfunction (EED) [4,5,6,7]. 

EED is a gut inflammatory asymptomatic condition that is characterized by T-cell infiltration of the intestinal mucosa, leading to chronic inflammation and blunting of the intestinal villi and thereby increasing intestinal permeability, translocation of gut microbes, micro- and macronutrient malabsorption, insufficient weight gain and stunted physical and cognitive development. While its etiology is poorly understood, poor hygiene and sanitation practices—particularly continuous exposure to fecal contamination—have been linked to the occurrence of EED [8]. The gold standard for diagnosing EED is an intestinal biopsy; however, this method is invasive and therefore not ideal [8]. The dual sugar absorption test is the most widely used marker of epithelial integrity. Other fecal and serum biomarkers such as fecal myeloperoxidase, calprotectin and neopterin are being studied for use in EED diagnosis. Like stunting and wasting, EED frequently occurs within the first three years of life, a period when infants transition from breastfeeding to complementary foods [8].

In traditional sub-Saharan African societies, complementary feeding is dominated by monotonous, low-quality protein and micronutrient-poor starchy foods such as maize, cassava, rice and sorghum. To meet recommended daily dietary intakes, use of such complementary foods require frequent feeding, about five meals per day, but the required resources in terms of cooking fuel and caretaking time are limited, making this option ineffective [3]. Dietary diversification is touted as an alternative, but in the face of general poverty this may not be an easy option for many rural communities to embrace, hence supplementation using locally available nutrient-dense foods may be considered a better option [3,9]. 

Protein-rich cereal legume blended flours such corn–soy blend (CSB) are the accepted standard dietary supplement in most LMICs where corn is the main staple. CSB is a fortified blended flour that contains selected micronutrients, sugar, maize and soy in different formulations depending on the manufacturer (typically 80:20 for corn and soy, respectively). CSB is used for prevention and treatment of acute malnutrition, wasting and growth retardation [10]. However, CSB requires central processing, is not readily available and accessible to most poor populations and its efficacy in improving linear growth remains debatable [11,12]. Other more readily available nutrient-dense legumes to supplement complementary foods should thus be explored. We supplemented the complementary diets of children aged 6–23 months for 6–12 months with common bean or cowpea flours as alternatives to CSB, and we used CSB flour as a control. In children aged 6–12 months, the cowpea flour added to the weaning food improved linear growth compared to common bean and CSB [13]; in 12–23-months-old children, the common bean supplement reduced lactulose excretion, a biomarker of gut health, but this did not result in better linear growth outcomes [14]. In a secondary analysis of these two cohorts, we found higher rates of stunting, wasting and EED, despite adequate macronutrient consumption in Masenjere, a region that had a higher consumption of energy, carbohydrates and fats, better food security and Water, Sanitation and Hygiene (WASH) outcomes than Limera [15].

More recently, research has indicated that dietary quality, especially protein quality, is important in assessing adequacy of diets to ameliorate the high rates of chronic and acute malnutrition in children consuming otherwise macro- and micronutrient-adequate diets [16,17,18]. Protein quality refers to the availability and digestibility of amino acids after food has been digested, absorbed and met the minimal obligatory rates of oxidation. Protein digestibility corrected amino acid score (PDCAAS) is currently the most used method of protein quality assessment. The PDCAAS method calculates the first limiting amino acid ratio in a gram of the target protein food against a reference protein, which this is then multiplied by the target protein’s digestibility factor, thereby predicting the body’s utilization of that protein. Other methods of protein quality assessment include digestible indispensable amino acid score (DIAAS), protein efficiency ratio (PER), plasma amino acid ratio, nitrogen balance, nitrogen requirements for humans and use of a reference protein [18]. Despite having a high protein content, plant-based diets have a low quality and low digestibility of proteins, as well as low amounts of bioavailable zinc, iron, calcium and other minerals. Animal-source foods (ASFs), on the other hand, provide better protein quality and bioavailability of vitamin B12, heme iron, vitamin A, zinc, calcium and other minerals [19]. Some studies and systematic reviews have demonstrated lower rates of stunting in children that consumed animal-source foods (fish, meat, poultry, dairy products, eggs), than children that only consumed plant-based diets [19,20,21,22,23,24,25,26].

In this secondary analysis, we hypothesized that the type of food and protein quality of food consumed by children from Masenjere and Limera villages would differ, despite a lack of significant variation in mean macronutrient intake between the two communities, and that this could explain the differences in rates of stunting, acute malnutrition and EED that were observed in the populations.

## 2. Materials and Methods 

We performed a secondary analysis of children who participated in two randomized controlled trials (RCTs) of common bean and cowpea flours addition as a part of the complementary food in Malawi. The details of the study protocols and principal clinical findings have been reported previously [13,14,27]. 

### 2.1. Primary Study Background

Briefly, these were prospective, double-blind, randomized controlled trials that we conducted in two rural southern Malawi villages: Limera village in Nsanje district, which is north of Blantyre district and has an elevation of 740 m; and Masenjere village in Machinga district, which is north-east of Blantyre district and has an elevation of 106 m. The children received isoenergetic amounts of common bean (*Phaseolus vulgaris*), cowpea (*Vigna unguiculata*), or CSB (control) flour to add to their usual complementary porridge. RCT 1 enrolled infants at 5.5–6.5 months of age who received the intervention for 6 months, while RCT 2 enrolled toddlers at 12–23 months of age who received the intervention for 12 months. Children who were of the required age and permanent residents of the study area were included for screening. All children that had severe or moderate malnutrition, congenital defects or gross developmental delay were excluded for screening. The aim of the original studies was to compare the efficacy of legume-based complementary food on infant and toddler linear growth and gut health in children at risk of developing EED. The primary outcome measure of these studies was change in height-for-age *z* score (HAZ), and changes in percentage of lactulose excretion (%L). The secondary outcomes included intervention effects on gut microbiome, other anthropometric indices, clinical morbidity and dietary intake assessment. The studies were registered at clinicaltrials.gov as NCT02472262 and NCT02472301.

### 2.2. Intervention Flours

Cowpea (Sudan 1 variety) and common bean (white variety) were sourced from local markets, roasted and milled into flour as previously described [28,29]. CSB was commercially produced by a local company (Rab Processors Limited, Blantyre, Malawi), and consisted of 70% corn and 30% soybean, with added sugar and fortified with vitamins A, B1, B2, B3 and B12, folate, calcium, zinc and iron. Each of the randomized interventions provided 30–40% of the child’s daily energy requirement from complementary food [28]. Using a plastic spoon that was provided to each participant (which delivered 10 g per scoop), guardians of the children were instructed to add the roasted common bean, cowpea or CSB flour to a serving of already-made porridge once a day every day for the duration of the study. Children were given 20 g, 30 g, 40 g and 50 g of flour at 6–8 months, 9–11 months, 12–23 months and 24–36 months, respectively.

### 2.3. Sample Calculation

In this secondary analysis we characterized food and nutrient intake in the two communities where the trials were conducted, Masenjere and Limera. A total of 355 children’s primary guardians provided 24-h dietary recalls conducted in either October 2015 or February 2016 (172 from RCT 1 and 183 from RCT 2). A post-hoc sample size analysis indicated that this sample had a power of 80% to detect an effect size of 0.48 in protein intake between the intervention and the control groups at a 0.05 significance level.

### 2.4. Interactive 24-h Dietary Recall

We used a multi-stage interactive 24-h dietary recall method that was specifically developed for use in LMICs with low literacy levels [30]; this method has been validated and modified for use in similar settings to our own [31,32,33,34,35]. Pre-testing of picture charts and development of a food list was done in earlier studies by the team [35,36]. The recall occurred over three consecutive days in four stages.

#### 2.4.1. Training the Primary Caregivers

Trained research assistants provided guardians with feeding utensils (a cup, bowl and spoon), pencils and picture charts featuring foods and drinks commonly consumed by the populations. The guardians were asked to use a tick to mark all items each child consumed the following day, and examples of effective recordings were demonstrated. The guardians were instructed to use only the provided utensils to feed the children the next day. There was no quantitative dimension to household recording, but the use of standardized utensils was intended to facilitate estimation of portion size during a follow-up interview (Stage 4).

#### 2.4.2. Recording Day

The next day, the guardians were instructed to feed children as normal, using the utensils provided, and to keep a record by ticking the picture chart. 

#### 2.4.3. Recording and Verification of Food Consumed

On the third day, the guardians attended the clinic with the picture chart. Research assistants asked questions about the items consumed by children the previous day, including the times that food and drinks were consumed. This information was filled into a questionnaire and subsequently compared to the picture chart to prompt and assess discrepancies during the interview. If differences existed between the picture chart and the questionnaire, the research assistant probed for more information. 

#### 2.4.4. Food Quantity and Nutrient Calculations 

Cooked salted food replicas and samples of commonly consumed foods were purchased and prepared from local markets and used to demonstrate quantities consumed. Using the provided utensils, guardians were first asked to place the amount of food or drink that was estimated to have been served to each child onto a calibrated digital scale. They were then asked to remove the amount of food or drink that was estimated to have been consumed, leaving behind any amount of food that was not eaten. Both amounts were measured, and the amount of food consumed was calculated as the difference in grams between the food served and food left behind during each feeding session. This was done for all the food and drink consumed within a 24-h period. The data was then entered into an Access database (Microsoft Corporation, Redmond, WA, USA) linked to a food composition table. The food composition table comprised individual and composite food items that were adjusted for nutrient losses from cooking where appropriate, using the United States Department of Agriculture (USDA) nutrient retention factors [37]. Nutrient values were derived from the Tanzanian [38], Mozambican [39] and West African food composition tables [40], manufacturers websites and the USDA nutrient database [41] by team members and previously published research [42]. From this food composition database, the consumed food was converted into nutrient intake for each participant.

### 2.5. Dietary Diversity Assessment

Dietary diversity, a measure of food intake that qualitatively assesses access to food variety and nutrient adequacy of an individual’s diet, was measured using the World health Organizations (WHO’s) infant and young child feeding practices (IYCFP) indicators. The guidelines consist of seven food groups: grains, roots and tubers, legumes and nuts, dairy products (milk, yogurt and cheese), flesh foods (meat, fish, poultry and organ meats), eggs, vitamin A-rich fruits and vegetables (red, yellow, orange and green fruits and vegetables) and other fruits and vegetables [43]. Minimum dietary diversity (MDD) is defined as the percentage of children 6–23 months of age who receive foods from four or more food groups. In this analysis, MDD was defined as the proportion of children 6–36 who consumed foods from ≥2 groups for 6–8 months, ≥3 groups for 9–11 months and ≥4 groups for 12–36 months; this has been previously used for Malawi. The rationale is that younger children consume less complimentary food due to breastfeeding unlike older children, hence this comparison is more appropriate [44]. The mean dietary diversity score (DDS) was calculated as the sum of the number of food groups consumed by each child divided by the total number of children who participated in the 24-h dietary recalls. A DDS of four or more is associated with good dietary quality, nutrient adequacy of diets and better access to food [43,45]

### 2.6. Protein Quality Assessment

To assess the protein quality of the diet of these children, we used the protein digestibility corrected amino acid score (PDCAAS), which determines the ability of a protein to meet the metabolic requirements for amino acids and nitrogen in the body. PDCAAS is calculated by multiplying the lowest amino acid ratio in the food or mixed diet by the true digestibility of the protein [46]. We used USDA food composition tables to get protein and indispensable amino acid (IAA) compositions of the food; the total amount of protein was then multiplied by each IAA and divided by the reference scoring pattern. The true fecal digestibility values of consumed foods were sourced from the Food and Agriculture Organization (FAO) protein quality evaluation report [18] and the FAO amino acid content of foods and biological data on proteins database [47]. A weighted-average protein digestibility of each child’s diet was calculated, and this was multiplied by the amino acid score to get the amino acid ratios. We used the 6 months to 3-years-old amino acid pattern as recommended by the FAO for reference scoring patterns [46]. The PDCAAS value for each child was then multiplied by the total protein intake of the child to determine the amount of bioavailable protein for use by the body.

### 2.7. EED Categorization

The full procedure for EED categorization has been previously published by the group [13,14,27]. Briefly, the dual sugar absorption method was used for categorizing EED; 20 mL of 1 g mannitol and 5 g lactulose solution was fed to the children after overnight fasting under the supervision of research personnel. Urine was then collected into collection bags/cups from the children for a minimum of 4 h after sugar ingestion. The total amount of urine collected per child was measured, aliquoted then flash frozen in liquid nitrogen for transfer to storage freezers. HPLC was used to quantify the sugars. A percentage of lactulose excretion (%L) detected in urine in relation to the amount ingested was measured. Children with %L < 0.2% were categorized as having no EED, while those with %L ≥ 0.2% were categorized as having EED. 

### 2.8. Statistical Analyses 

Because of the previous findings that the incidence of acute malnutrition (defined by a Mid-Upper Arm Circumference (MUAC) < 12.5, Weight for height Z-Score (WHZ) < −2, and presence of edema) was greater in Masenjere than Limera [15], the data were first disaggregated by location. All statistical analyses were conducted in Stata release 13 (Statacorp LLC, College Station, TX, USA). Post-hoc power calculation was done using G*Power version 3.0.1 (Erdfelder, Faul & Buchner, Dusseldorf, Germany). Baseline anthropometric calculations were based on WHO guidelines for child growth using Anthro version 3.2.2 (WHO). Wilcoxon rank sum test and Student’s *t*-test were used for non-normally distributed variables and normally distributed variables, respectively. Bivariate and multivariate analysis using linear regression were done to assess associations of the outcomes with potential confounders. The results were considered significant if the *p* values were less than 0.05.

### 2.9. Ethics Approval

The University of Malawi College of Medicine Research and Ethics Committee and the Washington University Human Research Protection Office provided the ethical approval to conduct this trial. District health officials and village heads from Machinga and Nsanje provided their support and consent for the study to be conducted within their areas prior to commencement of data collection.

## 3. Results

### 3.1. Baseline Characteristics of the Population

**Figure 1:**
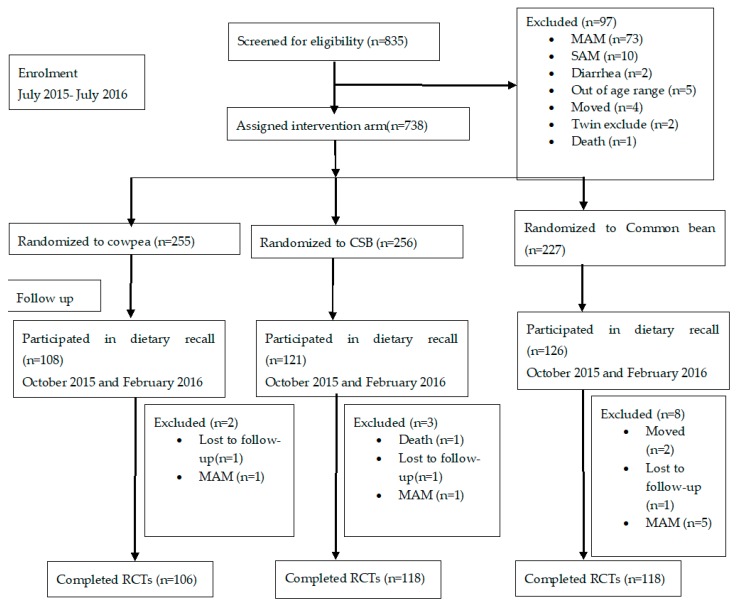
Flow chart of children aged 6–36 months that participated in the interactive 24-h dietary recall. MAM = moderate acute malnutrition; SAM = severe acute malnutrition; CSB = corn–soy blend; RCTs = randomized controlled trials.

A total of 355 children participated in the dietary recalls—172 from RCT 1, and 183 from RCT 2 (Figure 1). The characteristics of the study participants at baseline and at completion of the studies are reported in Table 1. In RCT 1, 100% of the children from Masenjere had access to improved water sources compared to only 34% of children from Limera, *p* < 0.001. A higher number of children from Masenjere slept in the same room as domesticated animals (chickens and goats) compared to children from Limera (43% vs. 15%, respectively, *p* < 0.001). At the end of the study, children from Limera had better weight-for-length *z* score (WLZ), length-for-age *z* score (LAZ) and weight-for-age *z* score (WAZ) compared to children in Limera, *p* < 0.001, *p* = 0.037 and *p* < 0001, respectively. There were 14% and 13% more children from Masenjere that were stunted and underweight compared to children from Limera, *p* = 0.048 and 0.003, respectively. No statistically significant results were observed for the other parameters.

In RCT 2, children from Limera had a higher mean weight, HAZ and WAZ, and a lower lactulose excretion than children from Masenjere, *p* = 0.041, *p* = 0.023, *p* = 0.027 and *p* = 0.034 at baseline, respectively. Children from Limera also had a higher number of siblings compared to those from Masenjere, *p* = 0.026. A higher number of children from Masenjere had access to an improved water source than children from Limera (99% vs. 45%, *p* < 0.001). At the end of the study, the change in WAZ was higher in children from Limera than Masenjere, *p* = 0.024. No statistically significant results were observed for the other parameters. 

### 3.2. Type of Food Consumed 

The foods, both composite and individual, consumed by children from both Masenjere and Limera are listed in Table 2. Chicken, avocado, cabbage, coca cola, egg broth, okra, oranges and tea were only consumed by children from Masenjere, while pawpaw, peas, white beans, sugar plum, guavas and icicles were only consumed by children from Limera; however, these foods were consumed by less than 20% of the children in both RCT 1 and 2. The total number of times a particular food was consumed was higher in Masenjere than Limera (55% vs. 46%, *p* < 0.001 in RCT 1 and 55 vs. 45, *p* < 0.001 in RCT 2). Fish was the most consumed animal-source food in both regions; a higher number of children from Limera consumed statistically significantly more fish than from Masenjere (54% vs. 35%, *p* = 0.009) in RCT 2. Solid meat was not consumed in either regions, only broth from meat stew was consumed, and this was statistically significantly higher in Masenjere than Limera (9% vs. 1%, *p* = 0.018) in RCT 1 but not in RCT 2 (1% vs. 0%, *p* = 0.313). In RCT 1, children from Limera consumed larger quantities of stiff maize porridge (nsima), red kidney beans and wheat flour fritters (mandasi) than children from Masenjere, leading to a statistically significant higher intake of energy (*p* = 0.015, *p* = 0.040 and *p* < 0.001) and protein (*p* = 0.023, *p* = 0.040 and *p* < 0.001), respectively, from these foods (Table A1). A higher consumption of protein was observed from maize porridge in children from Masenjere than from Limera, *p* = 0.0.001. In RCT 2, children from Masenjere consumed statistically significantly higher amounts of protein from maize porridge and mangoes than children from Limera, *p* = 0.042 and *p* = 0.022, respectively. 

### 3.3. Comparison of Dietary Diversity of the Children

The mean ± SD dietary diversity score (DDS) of the children’s diets was higher in Masenjere compared to Limera (2.8 ± 1.0 vs. 2.5 ± 1.1, *p* = 0.024), for RCT 2 but not RCT 1 (2.4 ± 1.2 vs. 2.3 ± 1.0, *p* = 0.442) (Table 3); this difference remained after adjusting for potential confounders (asset score, number of siblings, sex, age and season) in multivariate analysis (*p* = 0.018). Grains, roots, and tubers were the most consumed food group by the children (77–79% in RCT 1 and 83–89% in RCT 2), while dairy products were rarely consumed by this population (1% in RCT 1 and 0–3% in RCT 2) with differences between the two regions (*p* > 0.05). Children from Masenjere consumed more plant food groups, particularly legumes and nuts (32% vs. 12%, *p* = 0.001) and vitamin A-rich fruits and vegetables, than children from Limera (85% vs. 59%, *p* < 0.001) in RCT 2, and more vitamin A-rich fruits and vegetables (70% vs. 48%, *p* = 0.003) in RCT 1. Flesh foods, defined as intake of solid meat (excluding broth), were more consumed by children from Limera than Masenjere in both RCT 1 (62% vs. 38%, *p* = 0.001) and RCT 2 (59% vs. 43%, *p* = 0.032). Eggs were consumed more by children from Masenjere than children from Limera in both RCT 1 (12% vs. 4%, *p* = 0.048) and RCT 2 (17% vs. 5%, *p* = 0.017). More than one-third of the children in RCT 1 (37% in Masenjere and 42% in Limera) and more than half of the children in RCT 2 (78% in Masenjere and 83% in Limera) did not meet the recommended MDD; there was no difference between the two regions (*p* > 0.05). 

### 3.4. Comparison of Protein Quality in the Diets of Children

The mean PDCAAS values for children from Masenjere and Limera were 77.7% and 73.8%, respectively, in RCT 1, and 70.9% and 71.8%, respectively, in RCT 2, with Lysine as the limiting amino acid in both studies, reflecting a high consumption of grain staples. The food items consumed only in Masenjere had an average PDCAAS of 70% and 72% in RCT 1 and 2, respectively, contributing 12.9 g (2.0 g in RCT 1 and 6.9 g in RCT 2) of bioavailable protein. The food items consumed only in Limera had an average PDCAAS of 75% and 71% in RCT 1 and 2, respectively, contributing 12.0 g (6.2 g in RCT 1 and 5.8 g in RCT 2) of bioavailable protein, while the PDCAAS values between the two regions were similar (*p* = 0.773). The food consumed in Limera contributed 3.1 g more bioavailable protein than from Masenjere (*p* = 0.035). Animal-source protein consumption (defined as consuming chicken, fish or eggs at least once) was associated with a higher PDCAAS value compared to plant protein consumption, 63.7 ± 15.9 vs. 46.5 ± 6.0 (*p* < 0.001), respectively, in both regions. 

A higher percentage of children in Limera consumed animal-source food (defined as the intake of solid meat excluding broth) than in Masenjere, 4.9 ± 8.1 g/day vs 2.1 ± 4.2 g/day (*p* < 0.001), respectively, and mean quantities of animal-source food were higher in Limera compared to Masenjere (99(56%) vs. 79(44%), respectively, *p* = 0.030). Considering breastmilk consumption, children from Limera consumed 2.1 g/day more bioavailable protein from their total intake than children from Masenjere (*p* = 0.013) (Table 4). In children 12–36 months, no difference was observed for total protein consumption; however, children from Limera consumed higher amounts of bioavailable protein (*p* = 0.018). More than 80% of the children met their recommended daily protein intake, but the difference between the two regions tended toward significance in the 6–12-months-old group (*p* = 0.051) but not the 12–36-months-old group (*p* = 0.231) (Table 4). When ages were pooled, 87.6% and 93.2% of the children from Masenjere and Limera met their recommended levels of protein consumption, respectively; this was higher in Limera than Masenjere (*p* = 0.037). 

### 3.5. The Association of Food Type and Food Quality with Stunting, Acute Malnutrition and EED 

In bivariate analysis, children aged 6–12 months that consumed fish protein had a 0.3 increase in HAZ per month compared to children that did not consume any fish (*p* = 0.019). However, this association did not remain significant after adjusting for confounders in a multivariate analysis (*p* = 0.161). Meanwhile, data collection in October remained statistically significantly associated with a 0.3 increase in HAZ per month accounting for fish consumption, water quality, sex, age at recall, number of siblings, asset score, food security, stooling place and presence of animals in the child’s room (*p* = 0.048). The female gender and data collection in February were negatively associated with change in HAZ, −0.3 ± 0.1 (*p* = 0.046) and −0.4 ± 0.2 (*p* = 0.027), respectively, after adjusting for animal protein and other confounders (Table 5). Being female was associated with 0.1 increase in lactulose % excreted (*p* = 0.026). Consumption of animal-source food was not statistically significantly associated with lactulose % excreted (*p* = 0.437) or rate of acute malnutrition (*p* = 0.095) in both bivariate and multivariate analysis (Table 5). 

In children aged 12–36 months, a 1 g increase in consumption of animal-source protein was associated with a 0.02 increase in HAZ per month in bivariate (*p* = 0.021) and multivariate analyses (*p* = 0.047) (Table 5). In bivariate analysis, a 1 g higher intake of bioavailable protein was associated with a 0.02 increase in HAZ (*p* = 0.048). This association did not remain significant after adjusting for confounders; however, age remained statistically significantly associated with a 0.05 increase in HAZ per month after accounting for bioavailable protein, water quality, sex, season, number of siblings, asset score, food security, stooling place and presence of animals in the child’s room (*p* = 0.001). Age was significantly associated with a 0.1 increase in HAZ (*p* < 0.001) and a 0.01 increase in lactulose % excreted, after adjusting for animal protein (*p* = 0.003). Animal-source protein consumption was not associated with rates of acute malnutrition (*p* = 0.341) or lactulose % excretion (*p* = 0.437) (Table 5).

## 4. Discussion

In this secondary analysis, we assessed whether the diets of children from Masenjere and Limera differed, and if this difference was associated with different rates of stunting, acute malnutrition and EED observed in 6–36-months-old children within these communities. We have shown that children from Limera consumed a higher amount of protein from animal sources, and this was associated with improved linear growth in children aged 12–36 months. 

We have demonstrated that a region with lower rates of stunting, acute malnutrition and EED had a higher consumption of animal-source proteins than a region with lower consumption, despite equivalent or better nutrient intakes and sanitation practices. In our analysis, children from the Masenjere region consumed 2.1 g less mean bioavailable protein from animal sources, and consumption of animal-source protein was associated with improved linear growth in 12–36-months-old children. This agrees with findings from other studies. Krebs et al. [20] demonstrated a lower risk of stunting and wasting in children aged 5–24 months from the Democratic Republic of Congo, Zambia, Guatemala and Pakistan who consumed meat compared with those who did not. In Cambodia, consumption of animal-source foods was associated with lower rates of stunting (adjusted odds ratio of 0.69, *p* < 0.01) in children aged 12–59 months [21]. A study in Peru that assessed the breastfeeding and nutrient intakes of 12–15-months-old children demonstrated a 0.4 cm greater gain in child length over a 3-month period in weaned children that consumed food from animal products [51]. We speculate that a higher consumption of animal-source food in Limera was protective against worsening anthropometric outcomes in 12–36-months-old children but not 6–12-months-olds because older infants have lower ad lib breastmilk consumption and therefore consume more complementary food than children 6–12 months who consume more breastmilk than complementary food.

Fish was the main source of animal food in this population, followed by some consumption of eggs. Chicken, soup from beef and milk were consumed by very few children. While we have reported that 21–54% of the children consumed fish, the quantities of the fish consumed were small, providing mean energy intakes between 42–57 kcal/day and protein intakes of 3.9–5.5 g/day. Both Masenjere and Limera villages are located close to water bodies (Shire river and Lake Chilwa, respectively), hence there is an abundance of both small and big fish in the areas and it is cheaper than red meat or chicken. Because they are consumed with bones, organs and other viscera, small fish have a higher micronutrient content (especially of iron, zinc, calcium and vitamin A) compared to larger fish from which only the flesh is consumed [52]. Small fish are cheaper and more available on the market than large fish in Malawi, and it is therefore important to educate and encourage mothers to add more fish into the weaning diets of children. The use of dried fish and fish powder in complementary feeding programs is well established. A study in Ghana that assessed growth and micronutrient intake in children supplemented with a modified cereal–legume complimentary food called Weanimix found no differences in child growth for groups that received either Weanimix, fortified Weanimix, Weanimix plus fish powder or koko (a fermented corn meal or millet flour) plus fish powder. What is interesting about this finding is that the addition of fish seemed to have a protective effect on growth of children just as fortified Weanimix did, for consumption of koko alone has been linked to poor growth outcomes [53]. 

As expected, the diet of the children from both regions was predominantly plant based with maize staples as the most consumed food. This was also reflected in the low PDCAAS values with lysine as the limiting amino acid. While more than 80% of the children met their recommended daily protein intake, the quality of this protein was poor with low bioavailability, for it was mainly plant based. We have demonstrated a higher plant food consumption in Masenjere than Limera, and it is therefore not surprising that the region has poor growth outcomes since the overall quality of the diet is poor in quality bioavailable protein. The importance of consuming quality protein in infancy cannot be understated, as it impacts cognitive development. A Kenyan study that assessed the cognitive performance of school children found that children who received better nourishment (adequate diets with high energy, fat, carbohydrate and animal protein intake) had higher cognitive scores, played more and were more verbal compared to children with inadequate diets. Consumption of animal protein was more strongly associated with better cognitive outcomes than the intake of other nutrients. The study also established that intakes of animal protein between 18 and 30 months of age predicted higher cognitive scores at 5 years old [54]. Similarly, a Ghanaian study of healthy 6–9-years-old school children demonstrated higher cognitive scores in children that received 8.8 g milk protein per day compared with children that received 4.4 g milk protein per day or 4.4 g milk plus 4.4 g rice protein per day [55].

We have demonstrated that the quality of the nutrients consumed is more reflective of the observed nutrition status of children than meeting the minimum recommended dietary diversity. Children from Masenjere had a higher dietary diversity score, and more of them met the minimum required dietary diversity, which are indicators dietary adequacy; this did not, however, reflect in the anthropometric status of the children. In this study, a higher dietary diversity was not associated with improved growth outcomes and nutritional status. The positive association of higher dietary diversity with improved anthropometric outcomes has been reported by many and is an accepted indicator of dietary adequacy [44,56,57,58,59]. However, our findings suggest that assessing the source of the nutrients may be equally important in assessing the anthropometric outcomes of children. 

Some non-governmental organizations working within the regions of Masenjere and Limera recommend the addition of fish bones to maize for porridge flour preparation, but the uptake of this program is poor. Further studies investigating the feasibility and acceptability of adding dried fish to the complementary food of children in these regions are needed. 

A limitation of this study, as with all interactive 24-h dietary recalls, is that the number of recalls needed per individual to reduce uncertainty is about 10. Since this was not done in this work, our data can only be used to describe the collective populations of Masenjere and Limera. Modification of a child’s diet by the guardians is a potential source of bias in interactive 24-h recalls. In most cases, the guardians modified the diet of the child on the recall day to impress the researchers by adding foods that are not normally consumed. This could have led to over-reporting intake, which could have produced results that do not represent the usual intake amount. In this analysis, only 9 out of 70 (13%) children that developed acute malnutrition in the main RCTs 1 and 2 [15] participated in dietary recalls, so the association of food type and quality consumption with acute malnutrition may have been affected. More reflective results on this association would have been attained if we assessed the diet of all the children that developed acute malnutrition. 

## 5. Conclusions

We have demonstrated that consumption of animal-source protein is associated with an increase in height-for-age *z* scores in rural Malawian children aged 12–36 months. Animal-source food (ASF) is superior to plant-source food due to a rich protein and micronutrient profile, and we conclude that interventions that promote higher intakes of ASF in populations like this have the potential to reduce the incidence of stunting. Therefore, more efforts need to be made to promote the incorporation of these foods into the complementary foods of children. We acknowledge that ASF interventions are more expensive to implement, making their feasibility difficult in resource-poor settings, and we therefore suggest that further studies be conducted exploring the impact of alternative ASFs such as field mice and edible insects on growth outcomes. These alternative sources are cheaper and readily available in rural areas. Interventions to encourage fish farming in rural local communities would also help in improving ASF intake in both children and adults. 

## Figures and Tables

**Table 1:** Characteristics of 6–36-months-old Malawian children who participated in 24-h dietary recalls.

Characteristic at Enrolment	6–12 Months (*n* = 172)			12–36 Months (*n* = 183)		
	Masenjere (*n* = 87)	Limera (*n* = 85)	*p* Value	Masenjere (*n* = 91)	Limera (*n* = 92)	*p* Value
Girls, %	44	52	0.288	40	39	0.953
Age, months ^1^	5.8 ± 0.3	5.8 ± 0.3	0.537	17.6 ± 3.8	18.0 ± 3.7	0.582
Weight, kg ^1^	7.1 ± 0.7	7.1 ± 0.8	0.904	9.6 ± 1.3	10.0 ± 1.3	0.041
Length/height, cm ^1^	63.9 ± 2.6	63.6 ± 2.3	0.365	76.4 ± 5.4	77.9 ± 4.7	0.055
Mid-Upper Arm circumference (MUAC), cm ^1^	14.0 ± 0.9	14.1 ± 0.9	0.316	14.5 ± 0.8	17.8 ± 1.0	0.101
Weight-for-length/height *z* score (WLZ/H) ^1^	0.3 ± 0.9	0.4 ± 0.9	0.395	−0.1 ± 0.9	0.02 ± 0.9	0.329
Height/Length-for-age *z* score (H/LAZ) ^1^	−1.2 ± 1.1	−1.3 ± 0.9	0.536	−1.7 ± 1.3	−1.3 ± 0.1	0.023
Weight-for-age *z* score (WAZ)^1^	−0.6 ± 0.8	−0.5 ± 0.9	0.792	−0.9 ± 0.9	−0.6 ± 0.9	0.027
% Lactulose excreted	0.29 ± 0.2	0.25 ± 0.22		0.48 ± 0.32	0.39 ± 0.21	0.034
Stunted, %	25	20	0.408	37	30	0.322
Wasted, %	0	0	na	1	2	0.567
Underweight, %	1	5	0.165	12	4	0.056
Number of siblings ^1^	2.4 ± 1.9	2.6 ± 2.0	0.419	2.4 ± 1.8	3.0 ± 2.0	0.026
Asset score ^2^	1.2 ± 1.2	0.9 ± 0.9	0.062	1.1 ± 1.1	0.9 ± 0.9	0.129
Improved water source (borehole), %	100	34	<0.001	98.9	45	<0.001
Sleep with animals in bedroom, %	43	15	<0.001	32	23	0.191
Household severely food insecure, % ^3^	52	61	0.211	46	49	0.709
Characteristic at end of study						
Change in WLZ/H ^1^	−0.4 ± 0.9	0.01 ± 0.6	<0.001	−0.4 ± 0.8	−0.2 ± 0.7	0.080
Change in H/LAZ ^1^	−1.6 ± 0.9	−1.3 ± 0.8	0.037	−1.5 ± 0.8	−1.3 ± 0.9	0.084
Change in WAZ ^1^	−1.1 ± 0.7	−0.6 ± 0.7	<0.001	−1.1 ± 0.9	−0.8 ± 0.8	0.024
% Lactulose excreted	0.24 ± 0.17	0.21 ± 0.16	0.183	0.48 ± 0.25	0.47 ± 0.33	0.701
Stunted, %	41	27	0.048	30	33	0.668
Wasted, %	2	0	0.160	2	0	0.153
Underweight, %	17	4	0.003	21	5	0.002
Developed acute malnutrition, %	4	1	0.335	5	1	0.150

Student’s *t*-test was used for continuous variables and Chi-square test for categorical variables. ^1^ Values are means ± standard deviations. na= not available. ^2^ Asset score was defined as the sum of household items owned (radio, bicycle and iron sheet roof) with each item scoring 1 on the scale [48]. Improved water sources are defined as use of water from boreholes, since they are constructed to protect the water from outside contamination, particularly fecal matter [49]. ^3^ Severe food insecurity is defined as households that lacked resources to buy food, had no food in the home and went to sleep hungry one or more times per month [50]. na = not available.

**Table 2:** List of the type of foods consumed and their frequency of consumption by 6–36-months-old children from Masenjere and Limera villages.

Food Consumed	6–12 Months (*n* = 172)			12–36 Months (*n* = 183)		
	Masenjere (*n* = 87)	Limera (*n* = 85)	*p* Value	Masenjere (*n* = 91)	Limera (*n* = 92)	*p* Value
**Grains, roots and tubers**						
Maize on the cob, roasted	2	12	0.015	19	33	0.031
Maize porridge (watery), refined flour	72	82	0.120	68	71	0.595
Maize porridge (stiff, nsima), refined flour	81	86	0.342	95	89	0.185
Rice, boiled	1	0	0.322	6	9	0.399
Rice porridge, homemade	0	6	0.022	4	4	0.987
Cassava, boiled	0	0	na ^1^	1	8	0.031
Sweet potatoes, boiled	15	4	0.010	32	11	0.001
Fermented cereal drink (thobwa), homemade	26	5	<0.000	40	7	<0.001
**Flesh foods (meat, poultry, fish)**						
Fish, boiled	21	31	0.137	35	54	0.009
Soup, fish broth, homemade	13	41	<0.001	7	7	0.984
Soup, meat broth, homemade	9	1	0.018	1	0	0.313
Chicken, boiled	0	1	0.322	1	0	0.313
**Dairy products**						
Milk, powdered	1	1	0.987	3	0	0.079
**Eggs**						
Chicken egg, boiled/fried	10	4	0.079	17	5	0.017
Soup, egg broth, homemade	1	0	0.322	0	0	na ^1^
**Legumes and nuts**						
White beans, stewed	2	5	0.390	1	1	0.994
Cowpea, stewed	4	0	0.084	7	1	0.052
Red kidney bean, stewed	12	8	0.474	24	8	0.002
Pigeon pea, stewed	0	1	0.322	2	0	0.153
Peas, boiled	0	1	0.310	0	2	0.157
Soup, bean broth, homemade	2	2	0.981	1	0	0.313
**Vitamin A-rich fruits and vegetables**						
Mangoes, ripe	37	8	<0.001	43	22	0.002
Oranges, ripe	1	0	0.322	0	2	0.153
Pawpaw, ripe	0	0	na ^1^	0	1	0.319
Green leafy vegetables, boiled	59	72	0.071	74	45	<0.001
Guava, pink	0	0	na ^1^	0	3	0.082
Avocado, ripe, mashed	8	0	0.008	10	0	0.002
Okra, boiled	1	0	0.322	1	0	0.313
Soup from leafy vegetables	13	20	0.191	0	2	0.157
**Other fruits and vegetables**						
Cabbage, boiled	1	0	0.322	1	0	0.313
Banana, ripe	10	6	0.285	4	21	0.001
Sugar plum, ripe	0	2	0.150	0	3	0.082
Sugarcane, peeled	0	0	na ^1^	4	1	0.170
Snacks						
Concentrate juice, diluted	17	15	0.729	9	12	0.483
Kids’ maize snack, processed and colored (Kamba puffs)	33	9	<0.001	24	5	<0.001
Lollipop	14	9	0.190	41	17	<0.001
Cake, baked with banana, refined maize flour (chikondamoyo)	0	1	0.310	1	5	0.100
Fritters, fried with banana, refined maize flour (chitumbuwa)	1	0	0.310	4	21	0.001
Fritters, fried, refined wheat flour (mandasi)	14	12	0.690	29	16	0.047
Coca-cola	0	0	na ^1^	1	0	0.313
Icicles, sweetened and colored	0	1	0.310	0	1	0.319
Tea, unsweetened, brewed	2	0	0.160	3	0	0.079
Sugar	51	35	0.043	45	34	0.116

^1^ na = not available.

**Table 3:** Comparison of the dietary diversity of 6–36-months-old children from Masenjere and Limera.

Food Group	6–12 Months (*n* = 172)			12–36 Months (*n* = 183)		
	Masenjere (*n* = 87)	Limera (*n* = 85)	*p* Value	Masenjere (*n* = 91)	Limera (*n* = 92)	*p* Value
Grains, roots, and tubers, *n* (%)	79(91)	77(91)	0.961	88(97)	83(90)	0.076
Flesh foods (meat, poultry, fish), *n* (%)	33(38)	56(62)	0.001	39(43)	54(59)	0.032
Dairy products, *n* (%)	1(1)	1(1)	0.987	3(3)	0(0)	0.079
Eggs, *n* (%)	10(12)	3(4)	0.048	15(17)	5(5)	0.017
Legumes and nuts, *n* (%)	15(17)	12(14)	0.573	29(32)	11(12)	0.001
Vitamin A-rich fruits and vegetables, *n* (%)	61(70)	41(48)	0.003	77(85)	54(590	<0.001
Other fruits and vegetables, *n* (%)	11(13)	7(8)	0.345	6(7)	20(22)	0.003
Dietary diversity						
Number not meeting minimum dietary diversity (MDD) score, *n* (%)	32(37)	35(42)	0.555	71(78)	76(83)	0.435
Mean (SD) dietary diversity score	2.4 ± 1.2	2.3 ± 1.0	0.442	2.8 ± 1.0	2.5 ± 1.1	0.024

**Table 4:** Comparison of available protein for digestion in Malawian children aged 6–36 months with the addition of breastmilk.

Outcome	6–12 Months (*n* = 172)			12–36 Months (*n* = 183)		
	Masenjere (*n* = 87)	Limera (*n* = 85)	*p* Value	Masenjere (*n* = 91)	Limera (*n* = 92)	*p* Value
Total protein (g/day)	21.9 ± 8.7	24.3 ± 8.2	0.034	34.6 ± 13.1	37.1 ± 12.4	0.065
Bioavailable protein (g/day) ^1^	15.7 ± 5.8	16.7 ± 6.1	0.096	23.1 ± 8.1	26.0 ± 10.3	0.018
Recommended levels of intake (g/day) ^2^	11	11	na	13	13	na
% that met protein RDA	80.5	89.4	0.051	94.5	96.7	0.231

^1^ Bioavailable protein = PDCAAS X total protein. ^2^ Recommended levels of intake based on the WHO dietary reference intakes adopted from Borrensen et al. [28]. na = not applicable.

**Table 5:** Comparison of the association of Height-for-Age *z* score (HAZ), lactulose % and acute malnutrition, with covariates in children 6–36 months old who participated in 24-h dietary recalls using multiple linear regression.

	6–12 Months (*n* = 172)						12–36 Months (*n* = 183)					
	Change in HAZ		% Lactulose		Acute Malnutrition		Change in HAZ		Change in % Lactulose		Acute Malnutrition	
	Coef ± SE	*p* Value	Coef ± SE	*p* Value	Coef ± SE	*p* Value	Coef ± SE	*p* Value	Coef ± SE	*p* Value	Coef ± SE	*p* Value
Region (Limera vs. Masenjere)	−0.4 ± 0.2	0.062	0.7 ± 0.04	0.067	−0.01 ± 0.04	0.785	−0.2 ± 0.2	0.258	0.1 ± 0.04	0.088	−0.02 ± 0.03	0.469
Animal protein	0.01 ± 0.01	0.383	0.001 ± 0.002	0.437	0.00 ± 0.00	0.691	0.02 ± 0.01	0.047	−0.00 ± 0.00	0.317	0.00 ± 0.00	0.341
Improved water	0.3 ± 0.2	0.110	−0.02 ± 0.03	0.582	−0.01 ± 0.04	0.590	0.2 ± 0.2	0.206	−0.03 ± 0.04	0.389	0.02 ± 0.03	0.557
Sex (female vs. male)	−0.3 ± 0.1	0.046	0.1 ± 0.02	0.026	0.02 ± 0.02	0.394	−0.07 ± 0.1	0.638	0.1 ± 0.03	0.064	0.01 ± 0.02	0.657
Age	−0.1 ± 0.04	0.108	0.01 ± 0.01	0.222	0.00 ± 0.01	0.599	0.1 ± 0.01	<0.001	0.01 ± 0.00	0.003	0.00 ± 0.00	0.671
Number of siblings	0.02 ± 0.04	0.541	0.01 ± 0.01	0.053	na ^1^	na ^1^	−0.02 ± 0.04	0.592	−0.00 ± 0.01	0.722	−0.00 ± 0.01	0.617
Season (Feb vs. Oct)	−0.4 ± 0.2	0.027	0.05 ± 0.03	0.110	0.02 ± 0.03	0.576	0.04 ± 0.2	0.780	−0.01 ± 0.03	0.795	0.03 ± 0.03	0.292
Asset score (vs. 0)	0.03 ± 0.06	0.548	−0.01 ± 0.01	0.333	−0.02 ± 0.01	0.114	0.1 ± 0.1	0.362	0.00 ± 0.01	0.856	−0.01 ± 0.01	0.349
Severe food insecurity (vs. not)	0.1 ± 0.1	0.536	−0.02 ± 0.03	0.403	−0.04 ± 0.02	0.100	0.1 ± 0.2	0.641	0.04 ± 0.03	0.111	−0.00 ± 0.02	0.883
Stooling (pit latrine vs yard)	−0.1 ± 0.2	0.585	−0.04 ± 0.04	0.911	0.01 ± 0.04	0.713	0.1 ± 0.2	0.728	0.03 ± 0.04	0.427	0.01 ± 0.04	0.709
Sleep with animals	0.2 ± 0.2	0.178	−0.01 ± 0.03	0.631	−0.02 ± 0.03	0.547	0.1 ± 0.2	0.609	0.04 ± 0.3	0.178	0.00 ± 0.03	0.979

^1^ na = not available.

## Appendix A

**Table A1:** Comparison of the mean energy and protein intake from the food consumed by children aged 6–36 months.

Food consumed	6–12 Months						12–36 Months					
	^a^ Energy, Kcal/day ± SD			^b^ Protein g/day ± SD			^a^ Energy, Kcal/day ± SD			^b^ Protein g/d ± SD		
	Masenjer (*n* = 87)	Limera (*n* = 85)	*p* value	Masenjere (*n* = 87)	Limera (*n* = 85)	*p* value	Masenjere (*n* = 91)	Limera (*n* = 92)	*p* value	Masenjere (*n* = 91)	Limera (*n* = 92)	*p* value
**Grains, roots and tubers**												
Maize on the cob, roasted	14.6 ± 45.1	11.7 ± 12.5	0.779	0.5 ± 0.5	0.4 ± 0.4	0.779	40.7 ± 27.2	45.7 ± 28.7	0.556	1.4 ± 0.9	1.6 ± 1.0	0.556
Maize porridge (watery), refined flour	61.8 ± 34.6	61.0 ± 35.5	0.888	2.3 ± 2.4	1.4 ± 0.8	0.001	91.7 ± 50.8	98.7 ± 48.5	0.374	2.9 ± 3.0	2.2 ± 1.1	0.042
Maize porridge (stiff, nsima), refined flour	55.7 ± 33.2	66.0 ± 35.6	0.015	1.3 ± 0.7	1.5 ± 0.8	0.023	118.5 ± 66.2	121.9 ± 64.4	0.641	2.7 ± 1.5	2.7 ± 1.5	0.764
Rice, boiled	44.7 ± 0	0	na ^1^	0.8 ± 0	0	na ^1^	114.6 ± 77.6	252.4 ± 120.9	0.082	2.7 ± 1.4	4.7 ± 2.2	0.082
Rice porridge, homemade	0	34.2 ± 24.5	na ^1^	0	0.6 ± 0.5	na ^1^	161.7 ± 53.7	166.3 ± 81.9	0.928	2.7 ± 1.0	2.8 ± 1.3	0.966
Cassava, boiled	0	0	na ^1^	0	0	na ^1^	13.6 ± 0	55.7 ± 38.6	na ^1^	0.1 ± 0	0.5 ± 0.3	na ^1^
Sweet potatoes, boiled	38.0 ± 30.2	32.0 ± 7.2	0.746	0.7 ± 0.6	0.5 ± 0.1	0.587	67.8 ± 41.3	56.2 ± 26.4	0.411	1.1 ± 0.7	1.1 ± 0.6	0.416
Fermented cereal drink (thobwa), homemade	58.6 ± 42.9	32.7 ± 10.5	0.248	1.4 ± 1.1	0.7 ± 0.2	0.248	134.2 ± 104.8	99.8 ± 74.1	0.444	3.0 ± 2.3	2.2 ± 1.6	0.444
**Flesh foods (meat, poultry, fish)**												
Fish, boiled	42.8 ± 31.1	41.9 ± 45.3	0.928	3.9 ± 4.2	5.5 ± 6.8	0.312	44.5 ± 40.4	57.7 ± 50.5	0.171	4.2 ± 5.1	5.4 ± 6.4	0.307
Soup, fish broth, homemade	12.5 ± 4.5	13.7 ± 7.9	0.575	1.0 ± 0.4	1.1 ± 0.8	0.665	15.5 ± 8.4	11.8 ± 6.4	0.298	1.2 ± 0.6	1.1 ± 0.4	0.731
Soup. Meat broth, homemade	6.4 ± 3.1	9.4 ± 0	na ^1^	0.3 ± 0.1	0.4 ± 0	na ^1^	4.1 ± 0	0	na ^1^	0.2 ± 0	0	na ^1^
Chicken, boiled	15.4 ± 0	0	na ^1^	1.5 ± 0	0	na ^1^	54.0 ± 0	0	na ^1^	5.1 ± 0	0	na ^1^
**Dairy products**												
Milk, powdered	4.9 ± 0	9.8 ± 0	na ^1^	0.2 ± 0	0.5 ± 0	na ^1^	21.1 ± 1.4	0	na ^1^	1.02 ± 0.1	0	na ^1^
**Eggs**												
Chicken egg, boiled/fried	38.9 ± 23.7	49.1 ± 16.7	0.515	1.9 ± 0.8	2.8 ± 0.7	0.132	80.6 ± 56.5	73.7 ± 23.8	0.795	4.4 ± 2.3	4.3 ± 1.0	0.956
Soup, egg broth, homemade	7.0 ± 0	0	na ^1^	0.02 ± 0	2.8 ± 0.7	na ^1^	0	0	na ^1^	0	0	na ^1^
**Legumes and nuts**												
White beans, stewed	43.0 ± 5.7	0	na ^1^	0	1.7 ± 0.2	na ^1^	0	83.3 ± 0	na ^1^	0	3.4 ± 0	na ^1^
Cowpea, stewed	19.8 ± 11.9	0	na ^1^	0.9 ± 0.5	0	na ^1^	72.5 ± 49.1	49.5	0.570	3.3 ± 2.2	2.2 ± 1.4	0.551
Red kidney bean, stewed	26.1 ± 9.7	45.6 ± 29.8	0.040	1.4 ± 0.6	2.4 ± 1.5	0.040	62.3 ± 38.4	64.0 ± 29.6	0.909	3.3 ± 2.0	3.5 ± 1.6	0.825
Pigeon pea, stewed	0	40.9 ± 0	na ^1^	0	1.8 ± 0	na ^1^	70.6 ± 78.1	0	na ^1^	3.3 ± 3.2	0	na ^1^
Peas, boiled	0	0	na ^1^	0	0	na ^1^	0	39.2 ± 21.9	na ^1^	0	0.9 ± 0.5	na ^1^
Soup, bean broth, homemade	9.5 ± 0.5	5.6 ± 3.3	0.240	0.2 ± 0.01	0.3 ± 0.3	0.864	5.6 ± 0	0	na ^1^	0.3 ± 0	0	na ^1^
**Vitamin A-rich fruits and vegetables**												
Mangoes, ripe	63.6 ± 41.2	40.9 ± 14.4	0.159	0.5 ± 0.3	0.3 ± 0.1	0.146	127.3 ± 72.6	75.3 ± 42.4	0.003	1.0 ± 0.6	0.5 ± 0.3	0.002
Oranges, ripe	9.7 ± 0	0	na ^1^	0.2 ± 0	0	na ^1^	22.5 ± 0.9	0	na ^1^	0.4 ± 0.01	0	na ^1^
Pawpaw, ripe	0	0	na ^1^	0	0	na ^1^	0	19.5 ± 0	na ^1^	0	0.3 ± 0	na ^1^
Green leafy vegetables, boiled	12.0 ± 8.1	12.1 ± 12.0	0.968	0.3 ± 0.3	0.4 ± 0.5	0.275	26.7 ± 23.2	25.0 ± 21.8	0.662	0.8 ± 0.8	0.6 ± 0.6	0.105
Guava, pink	0	0	na ^1^	0	0	na ^1^	0	28.1 ± 27.0	na ^1^	0	0.4 ± 0.4	na ^1^
Avocado, ripe, mashed	74.6 ± 24.5	0	na ^1^	0.9 ± 0.3	0	na ^1^	91.2 ± 39.5	0	na ^1^	1.1 ± 0.5	0	na ^1^
Okra, boiled	74.6 ± 24.5	0	na ^1^	0.01 ± 0	0	na ^1^	2.2 ± 0	0	na ^1^	0.1 ± 0	0	na ^1^
Cabbage, boiled	7.6 ± 0	0	na ^1^	0.1 ± 0	0	na ^1^	63.4 ± 0	0	na ^1^	1.7 ± 0	0	na ^1^
Banana, ripe	26.7 ± 18.4	35.1 ± 3.8	0.342	0.3 ± 0.2	0.4 ± 0.04	0.342	40.6 ± 23.4	44.5±26.2	0.789	0.4 ± 0.3	0.5 ± 0.3	0.789
Sugar plum, ripe	0	2.3 ± 0	na ^1^	0	0.01 ± 0	na ^1^	0	2.4 ± 1.0	na ^1^	0	0.02 ± 0.01	na ^1^
Sugarcane, peeled	1.3 ± 0	0	na ^1^	0	0	na ^1^	6.2 ± 0.6	4.7 ± 0	na ^1^	0	0	na ^1^
**Snacks**												
Concentrate Juice, diluted	32.1 ± 25.3	33.1 ± 17.2	0.901	0.1 ± 0.1	0.1 ± 0.1	0.891	49.6 ± 50.7	75.3 ± 50.9	0.282	0.1 ± 0.1	0.2 ± 0.2	0.338
Kids maize snack, processed and colored (kamba puffs)	74.9 ± 100.6	53.7 ± 49.4	0.566	0.8 ± 1.0	0.6 ± 0.5	0.566	50.3 ± 44.3	66.7 ± 73.3	0.517	0.5 ± 0.5	0.7 ± 0.8	0.517
Lollipop	36.4 ± 31.1	35.8 ± 0	0.958	0	0	na ^1^	39.6 ± 13.9	40.3 ± 12.2	0.861	0	0	na ^1^
Cake, baked with banana, refined maize flour (chikondamoyo)	0	50.5 ± 0	na ^1^	0	1.4 ± 0	na ^1^	148.7 ± 0	131.7 ± 43.6	na ^1^	4.3 ± 0	3.8 ± 1.2	na ^1^
Fritters, fried with banana, refined maize flour (chitumbuwa)	0	531.1 ± 0	na ^1^	0	15.2 ± 0	na ^1^	213.3 ± 110.2	186.4 ± 130.8	0.703	6.1 ± 3.2	5.3 ± 3.7	0.703
Fritters, fried, refined wheat flour (mandasi)	168.4 ± 129.4	365.1 ± 104.7	<0.001	1.8 ± 1.4	3.9 ± 1.1	<0.001	380.1 ± 142.4	446.5 ± 255.3	0.285	4.1 ± 1.5	4.8 ± 2.7	0.285
Coca-Cola	0	0	na ^1^	0	0	na ^1^	71.3 ± 0.5	0	na ^1^	0	0	na ^1^
Icicle, sweetened and colored	0	95.5 ± 0	na ^1^	0	0.2 ± 0	na ^1^	0	33.0 ± 0	na ^1^	0	0.1 ± 0	0.379
Tea, unsweetened, brewed	11.8 ± 20.5	0	na ^1^	0.4 ± 0.7	0	na ^1^	13.7 ± 12.6	0	na ^1^	0	0	na ^1^
Sugar	40.3 ± 21.4	48.1 ± 20.3	0.073	0	0	na ^1^	55.6 ± 25.1	60.5 ± 22.9	na ^1^	0	0	na ^1^

^a^ Energy kcal/day ± SD: kcal/day is the mean energy consumed per day. ^b^ Protein g/day ± SD: g/day is the mean amount of protein provided by each food per day. ^1^ na = not available.
